# First Phase I human clinical trial of a killed whole-HIV-1 vaccine: demonstration of its safety and enhancement of anti-HIV antibody responses

**DOI:** 10.1186/s12977-016-0317-2

**Published:** 2016-11-28

**Authors:** Eunsil Choi, Chad J. Michalski, Seung Ho Choo, Gyoung Nyoun Kim, Elizabeth Banasikowska, Sangkyun Lee, Kunyu Wu, Hwa-Yong An, Anthony Mills, Stefan Schneider, U. Fritz Bredeek, Daniel R. Coulston, Shilei Ding, Andrés Finzi, Meijuan Tian, Katja Klein, Eric J. Arts, Jamie F. S. Mann, Yong Gao, C. Yong Kang

**Affiliations:** 1 Department of Microbiology and Immunology, Schulich School of Medicine and Dentistry, The University of Western Ontario, 1400 Western Road, London, ON N6G 2V4 Canada; 2 Sumagen Canada Inc., London, ON Canada; 3 Anthony M. Mills Internal Medicine, West Hollywood, CA USA; 4 Long Beach Education and Research Consultants, Long Beach, CA USA; 5 Metropolis Medical PC, San Francisco, CA USA; 6 Rockwood Clinics, P.C., Spokane, WA USA; 7 Centre de Recherche du CHUM, Department of Microbiology, Université de Montréal, Montreal, QC Canada

**Keywords:** AIDS, HIV, Killed whole-HIV vaccine, Safety, Clinical trial, Immune responses, Neutralizing antibodies

## Abstract

**Background:**

Vaccination with inactivated (killed) whole-virus particles has been used to prevent a wide range of viral diseases. However, for an HIV vaccine this approach has been largely negated due to inherent safety concerns, despite the ability of killed whole-virus vaccines to generate a strong, predominantly antibody-mediated immune response in vivo. HIV-1 Clade B NL4-3 was genetically modified by deleting the *nef* and *vpu* genes and substituting the coding sequence for the Env signal peptide with that of honeybee melittin signal peptide to produce a less virulent and more replication efficient virus. This genetically modified virus (*gm*HIV-1_NL4-3_) was inactivated and formulated as a killed whole-HIV vaccine, and then used for a Phase I human clinical trial (Trial Registration: Clinical Trials NCT01546818). The *gm*HIV-1_NL4-3_ was propagated in the A3.01 human T cell line followed by virus purification and inactivation with aldrithiol-2 and γ-irradiation. Thirty-three HIV-1 positive volunteers receiving cART were recruited for this observer-blinded, placebo-controlled Phase I human clinical trial to assess the safety and immunogenicity.

**Results:**

Genetically modified and killed whole-HIV-1 vaccine, SAV001, was well tolerated with no serious adverse events. HIV-1_NL4-3_-specific PCR showed neither evidence of vaccine virus replication in the vaccine virus-infected human T lymphocytes in vitro nor in the participating volunteers receiving SAV001 vaccine. Furthermore, SAV001 with adjuvant significantly increased the pre-existing antibody response to HIV-1 proteins. Antibodies in the plasma of vaccinees were also found to recognize HIV-1 envelope protein on the surface of infected cells as well as showing an enhancement of broadly neutralizing antibodies inhibiting tier I and II of HIV-1 B, D, and A subtypes.

**Conclusion:**

The killed whole-HIV vaccine, SAV001, is safe and triggers anti-HIV immune responses. It remains to be determined through an appropriate trial whether this immune response prevents HIV infection.

## Background

Despite the tremendous advances in immunology and molecular biology accomplished since the discovery of HIV-1 [1], results of vaccine trials against HIV have remained extremely poor. Only one trial out of more than one hundred showed a modest protection while all the others did not induce any protective immunity against the virus [2–7]. Overall there is, therefore still a need to propose unexplored avenues. Here, we decided to explore the use of a full inactivated virus as immunogen, the most classical vaccine track [8–13] that has, however, never been adequately studied thus far, since the only attempt has been that of Jonas Salk with his Remune [14] which has been shown to have lost its envelope glycoprotein.

Vaccination with whole, inactivated (killed) virus particles has been used to prevent a wide range of viral diseases [8–13]. However, for inherent safety concerns, this approach has been largely negated for HIV-1 vaccine despite the ability of inactivated but intact whole-virus vaccines to generate a strong, predominantly antibody-mediated immune response in vivo. Previous developments in inactivation methods have greatly enhanced the utility of these immunogens [11, 15–18]. These chemicals allow elimination of virus infectivity to undetectable levels while maintaining the native protein conformation, including that of the important HIV-1 viral envelope surface glycoprotein (gp120) which is the major target for neutralizing antibody response in vivo [19, 20]. The killed whole-HIV-1 vaccine approach has great merit as it has the potential to present multiple viral antigens to the immune system in their native conformations. Thus, several scientists have recently suggested that it is time for another look at inactivated (killed) HIV vaccine for prevention of HIV infection [21, 22]. Interestingly, only one group (Remune) previously attempted a killed whole-HIV vaccine. However, lack of gp120 on the virion surface and other safety concerns led to the discontinuation of their project [14].

The challenges in developing a killed whole-HIV vaccine include safety issues associated with virus production and completely “killing” the virus on a large scale. In the present study, we demonstrate the result of our recently conducted Phase I human clinical trial for safety evaluation of our killed whole-HIV-1 vaccine, SAV001. The primary objective of this study was to evaluate the safety and tolerability of a single dose of SAV001 (with or without adjuvant, Montanide^®^ISA51VG) administered intramuscularly in men and women with chronic HIV-1 infection who were on cART. As a secondary measure, we also evaluated the vaccine-elicited humoral immune responses against the structural proteins of HIV-1 including the neutralizing antibody activity in the vaccinated individuals.

## Methods

### Construction of *gm*HIV-1_NL4-3_ virus

**Fig. 1:**
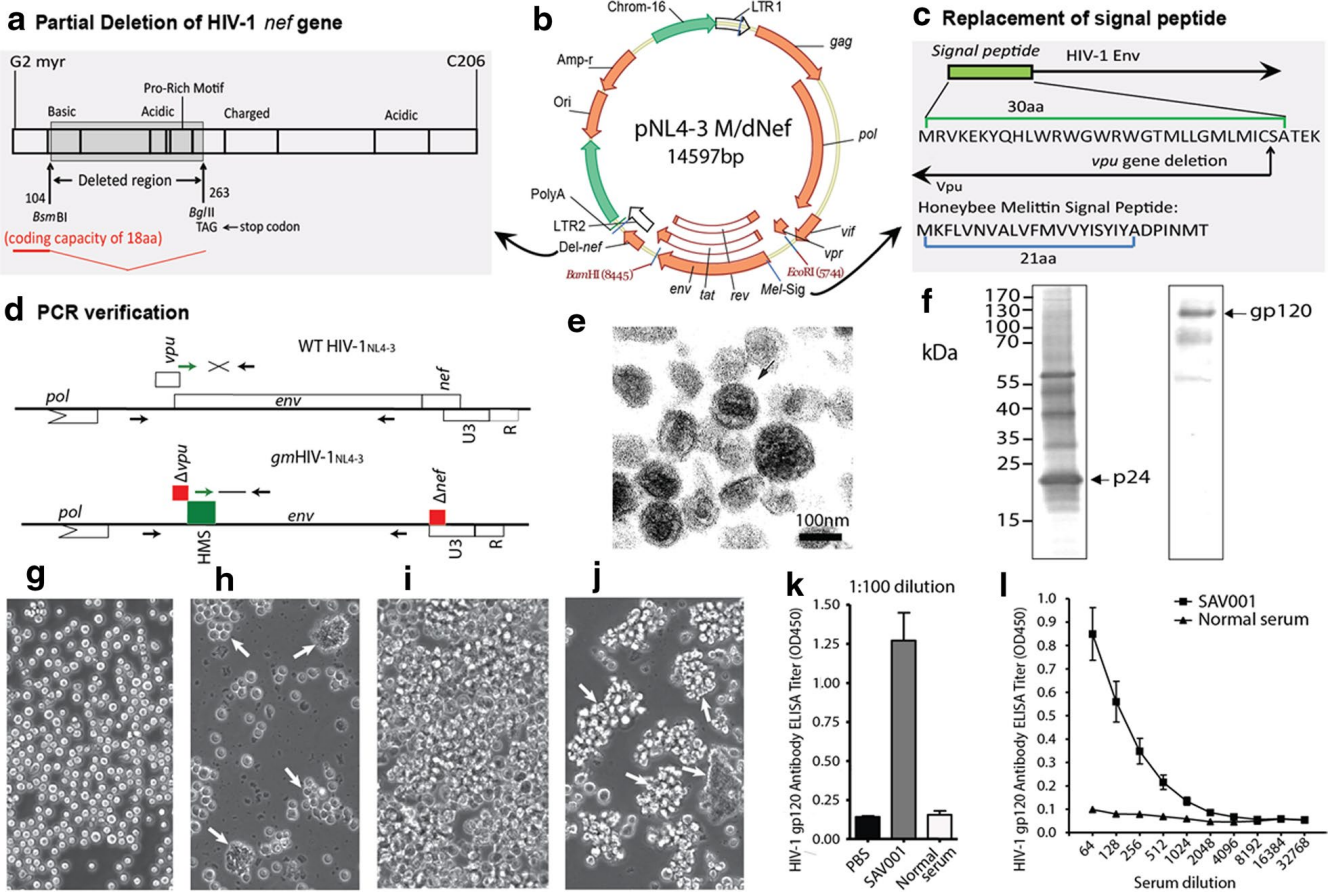
Construction of *nef*-, *vpu*-, and glycoprotein signal peptide replaced HIV-1_NL4-3_ (*gm*HIV-1_NL3-4_) and characterization of the SAV001 vaccine virus: The fragment between *Bsm*BI and *Bg*lII (from 104 to 263 nucleotides) of HIV-1 NL4-3 *nef* gene was deleted and the stop codon (TAG) was inserted (**a**). The coding region of HIV-1 *env* signal peptide (30 amino acids) was replaced by the coding sequence of honeybee melittin signal peptide (21 amino acids) [24] (**c**). The *vpu* gene was deleted as the result of the Env signal peptide gene replacement due to its overlapping reading frame in the upstream of *env* gene. The pNL4-3 M/dNef with *env* signal peptide replaced plasmid was transfected into A3.01 human T-lymphocytes and recovered the genetically modified HIV-1 NL4-3 (*gm*HIV-1_NL4-3_) virus (**b**). The genetic modification was confirmed by RT-PCR using honeybee melittin-specific primer with modified *nef* gene-specific primer (**d**). Electron micrograph of SAV001 vaccine showing intact virion (**e**). Western blot analyses of SAV001 using HIV-1 p24 antiserum (The NIH AIDS Reagent Program) and goat anti-HIV-1 gp120 polyclonal antibody (BIODESIGN), respectively (**f**). Aggregation of CD4^+^ AA2 [**g**, **h** (*arrow*)], and A3.01 [**i**, **j** (*arrow*)], as a result of gp120 on the SAV001 vaccine virus binding to the cell surface receptors. Induction of anti-gp120 antibody in SAV001 vaccine immunized rat sera using the gp140 trimer by ELISA (**k**, **l**)

The *nef* **-**, *vpu* **-**, and the Env-signal peptide replaced the virus used in this study were generated from the HIV-1 Clade B infectious molecular clone, pNL4-3 (The NIH AIDS Reagent Program). The fragment between *Bsm*BI and *Bg*lII (159 base pairs from 104 to 263 nucleotides) in the upstream of *nef* gene (modified from Flint et al. [23]) was deleted and the stop codon (TAG) was inserted in order to generate attenuated HIV-1. The coding region of HIV-1 *env* signal peptide (30 amino acids) was replaced by the coding sequence of honeybee melittin signal peptide (21 amino acids) [24] in order to increase the efficiency of this genetically modified HIV-1 replication. The *vpu* gene was deleted as the result of the Env signal peptide gene replacement due to its overlapping reading frame in the upstream of *env* gene (Fig. 1). The pNL4-3 M/dNef with *env* signal peptide replaced plasmid was transfected into A3.01 human T-lymphocytes and recovered the genetically modified HIV-1 NL4-3 (*gm*HIV-1_NL4-3_) virus. To confirm the genetic modification we RT-PCR amplified the SAV001-specific viral RNA using a primer specific for the honeybee melittin signal peptide coding sequence.

### Characterization of *gm*HIV_NL4-3_ virus

The electron microscopy was carried out by placing *gm*HIV-1_NL4-3_ virus in SAV001 vaccine onto a carbon grid, negatively stained with 1% PTA, and then examined in a Philip EM300 transmission electron microscope. Western blot analyses using HIV-1 p24 antiserum (The NIH AIDS Reagent Program) and goat anti-HIV-1 gp120 polyclonal antibody (BIODESIGN), respectively, carried out by the standard procedure to show the presence of capsid protein p24 and gp120 glycoprotein on SAV001 vaccine. Aggregation of CD4^+^ human T cell lines was examined by fluorescent microscopy after addition of SAV001 vaccine virus to CD4^+^ human T cell lines, AA2 and A3.01. Cultured AA2 and A3.01 cells were incubated with SAV001 vaccine virus for 30 min and examined under fluorescent microscope. To demonstrate that intact gp120 is present on the *gm*HIV-1_NL4-3_ (SAV001) vaccine virus, five 8 week-old Wistar rats per group were prime-immunized with 20 µg of SAV001 vaccine which was mixed 1:1 with Montanide. The rats were boost-immunized with the same concentration of the SAV001 three weeks after the prime-immunization. Blood from the immunized rats was collected one week after the boost-immunization and gp140 trimer was used as an antigen (The NIH AIDS Reagent Program) to detect gp120-specific antibodies in the immunized rat sera by ELISA.

### GMP manufacturing of SAV001

The killed whole-*gm*HIV-1_NL4-3_ was manufactured in the cGMP facility of Omnia Biologics, Inc. (Rockville, MD, USA) with total 120 l scale. The infectious *gm*HIV-1_NL4-3_ virus titer was measured by TCID_50_ assay using AA-2 human T lymphocyte. The infected culture fluid was inactivated, concentrated and purified by sucrose gradient ultracentrifugation. SAV001 was released for Phase I clinical trial based on the results of purity, identity, sterility, and safety tests under the GLP conditions.

### Inactivation processes of *gm*HIV-1_NL4-3_ virus

Double inactivation procedures were used for completely killing *gm*HIV-1_NL4-3_ virus during GMP manufacturing processes, respectively. The purified viruses from harvest underwent a chemical inactivation step by incubating the virus with a 2.5 mM solution of 2-2′-dithiodipyridine (aldrithiol-2 or AT-2) for 2 h at 37 °C with shaking.

Gamma Irradiation was performed off-site at STERIS Isomedix services at the end of manufacturing process. Grid boxes containing filled vials were irradiated via a Cobalt-60 source, receiving a dose of 30 KGy (3,000,000 rads) of gamma radiation (Table 1).

**Table 1:** Inactivation steps in virus production and the log reduction at each inactivation step
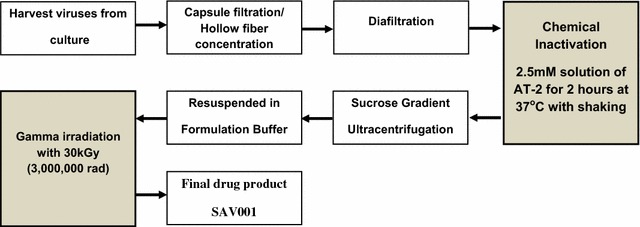

Inactivation method	Log_10_ reduction	Master virus bank (Log_10_TCID_50_/ml)	Putative safety margin
AT-2	≥4.83 ± 0.26	5.64 ± 0.10	≥4.71 log units
Gamma irradiation	≥5.52 ± 0.33		

### Study vaccine, adjuvant, and placebo

Genetically modified *gm*HIV-1_NL4-3_ virus (Fig. 1) was propagated in A3.01 human T-cell line, purified through the down-stream purification procedures and inactivated with a 2.5 mM solution of aldrithiol-2 (AT-2) for 2 h at 37 °C followed with γ-irradiation with a Cobalt-60 source, exposing a dose of 30 kGy (3,000,000 rads) of γ-radiation. Samples were tested through >230 product release assays at the GLP facilities to measure any residual virus replication or impurities according to the US FDA mandated product release tests. SAV001 was supplied in a 400 µl aliquot containing 160 µg (100 µg/0.25 ml) of total *gm*HIV-1_NL4-3_ virus protein which was then formulated with 75 mM NaCl, 25 mM HEPES pH7.4 with 5% trehalose mixed with an equal volume of adjuvant, Montanide^®^ISA51VG (Seppic, France). Montanide^®^ISA51VG is an oil-in-water emulsion. The surfactant mannide monooleate in Montanide^®^ISA51VG contains vegetable-grade oleic derived from olive oil [25]. Preservative-free sterile isotonic saline (0.9%) was used as the placebo. The Phase I human clinical trial of SAV001 was cleared by the US FDA for human clinical trials (Trial Registration: Clinical Trials NCT01546818).

### Study design and population

**Fig. 2:**
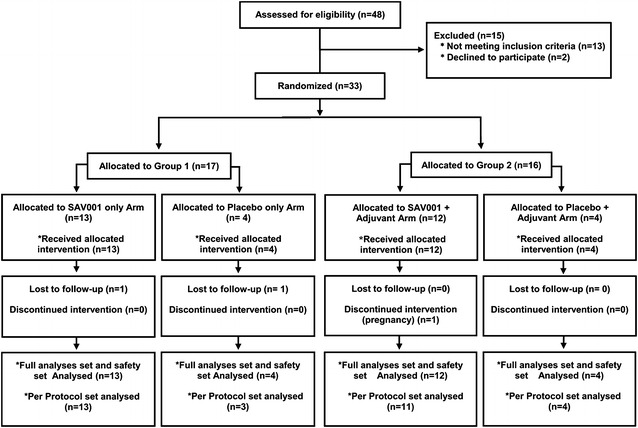
Flow diagram. The study was a randomized, observer-blinded, placebo-controlled study of killed-whole HIV-1 vaccine (SAV001-H) with or without adjuvant administered intramuscularly. Patients were screened for participation in this study within 28 days before enrollment. A total of 33 patients were planned to be enrolled in this study and randomized into Groups 1 and 2 of 17 and 16 patients, respectively. Patients were randomly assigned in a 9:3 ratio to receive either a single injection of the active investigational product or placebo, each of which was administered either without (Group 1) or with (Group 2) adjuvant

This study was randomized, observer-blinded, and placebo-controlled with or without adjuvant administered intramuscularly as a single dose. A total of thirty-three (n = 33) subjects enrolled into the study and were randomly assigned to groups (Fig. 2). The subjects were prescreened and eligibility evaluated for the study. After a 28 day screening period, subjects were randomized into 2 groups. The Full Analysis Set (FAS) and the Safety Set included 33 subjects (13 in the SAV001 group, 4 in the Placebo group, 12 in the SAV001 + Adjuvant group, and 4 in the Placebo + Adjuvant group; Table 2). The major reasons for screen failure were renal dysfunction, higher plasma HIV-1 RNA level, or lower CD4^+^ T cell counts.

**Table 2:** Characteristics of the study subjects

Study group	Subject ID	Age/sex/race	Infected HIV-1 subtype	Days of cART prior to entry	CD3 +/CD4 + level at entry (cells/µl)	Plasma HIV-1 RNA level at entry (copies/ml)
SAV001	2001004	43/male/black	B	537	1826	<2.00E+1
	1001005	37/male/white	ND	443	570	<2.00E+1
	2001008	40/male/white	B	1380	830	<2.00E+1
	2001009	28/male/white	A	960	500	<2.00E+1^d^
	2001012	27/male/white	B	1832	598	<2.00E+1
	3001015	35/male/white	B	1185^c^	694	2.50E+1
	3001018	44/male/white	B	1430	804	<2.00E+1
	2001022	30/male/white	B	229	419	<2.00E+1
	3001024	50/male/white	B	1097	486	2.22E+1
	1001025	44/female/white	B	2478	574	<2.00E+1
	4001029	30/male/black	B	445	491	<2.00E+1
	3001032	46/female/other^a^	ND	1885^c^	440	<2.00E+1
	3001033	45/male/white	B	395^c^	515	<2.00E+1
Placebo	2001002	25/male/white	B	1011	702	<2.00E+1
	2001013	46/male/white	ND	573	618	<2.00E+1
	1001020	48/male/white	B	1843	754	<2.00E+1
	3001027	25/male/white	B	265	366	<2.00E+1
SAV001 + adjuvant	1001001	46/male/white	B	1269	539	<2.00E+1
	1001003	49/male/white	A	1289	833	<2.00E+1
	2001006	21/male/white	B	333	581	<2.00E+1
	2001010	46/male/white	B	4745^c^	739	<2.00E+1
	2001011	33/female/black	B	1559	369	<2.00E+1
	3001016	48/male/white	B	730^c^	626	<2.00E+1
	3001017	43/male/white	B	1550	459	<2.00E+1
	3001019	35/male/Asian	A	2220^c^	555	<2.00E+1
	2001021	49/male/white	B	1848	1079	<2.00E+1
	3001026	29/male/white	C	252	485	<2.00E+1
	4001028	41/male/black	A	637	449	<2.00E+1
	4001031	40/male/white	ND	1290	794	<2.00E+1
Placebo +adjuv.	1001014	47/male/white	B	970^c^	827	<2.00E+1
	2001007	48/male/asian	D	638	498	<2.00E+1
	1001023	49/male/white	B	1316	1057	<2.00E+1
	4001030	25/female/other^b^	B	448	519	<2.00E+1

*ND* not done

^a^Native American and black

^b^Mexican

^c^Approximate days

^d^Evaluated at screening day

The study population consisted of adult males (n = 29) and females (n = 4) with chronic HIV infection who were undergoing treatment with cART, viral load with HIV-1 RNA levels of <75 copies/ml, and CD4^+^ T-cell count of at least 350 cells/μl for at least 6 months. Female subjects underwent a serum or urine pregnancy test before vaccination. Sexually active subjects agreed to use an effective method of contraception from the day of vaccination for four months.

In group 2, SAV001 or saline was mixed with the adjuvant prior to injection at the site in a 1:1 vol/vol ratio (0.25 ml each) for a final injection volume of 0.5 ml/dose. Because of solution appearance differences, 100 µg of SAV001 was administered by an unblinded member and ensured that investigator and subject remained blinded.

The study was conducted with prior written approval of a properly constituted institutional review board. Informed consent was obtained from each subject according to regulatory and legal requirements.

### Determination of residual infectious virus in SAV001 vaccine

**Fig. 3:**
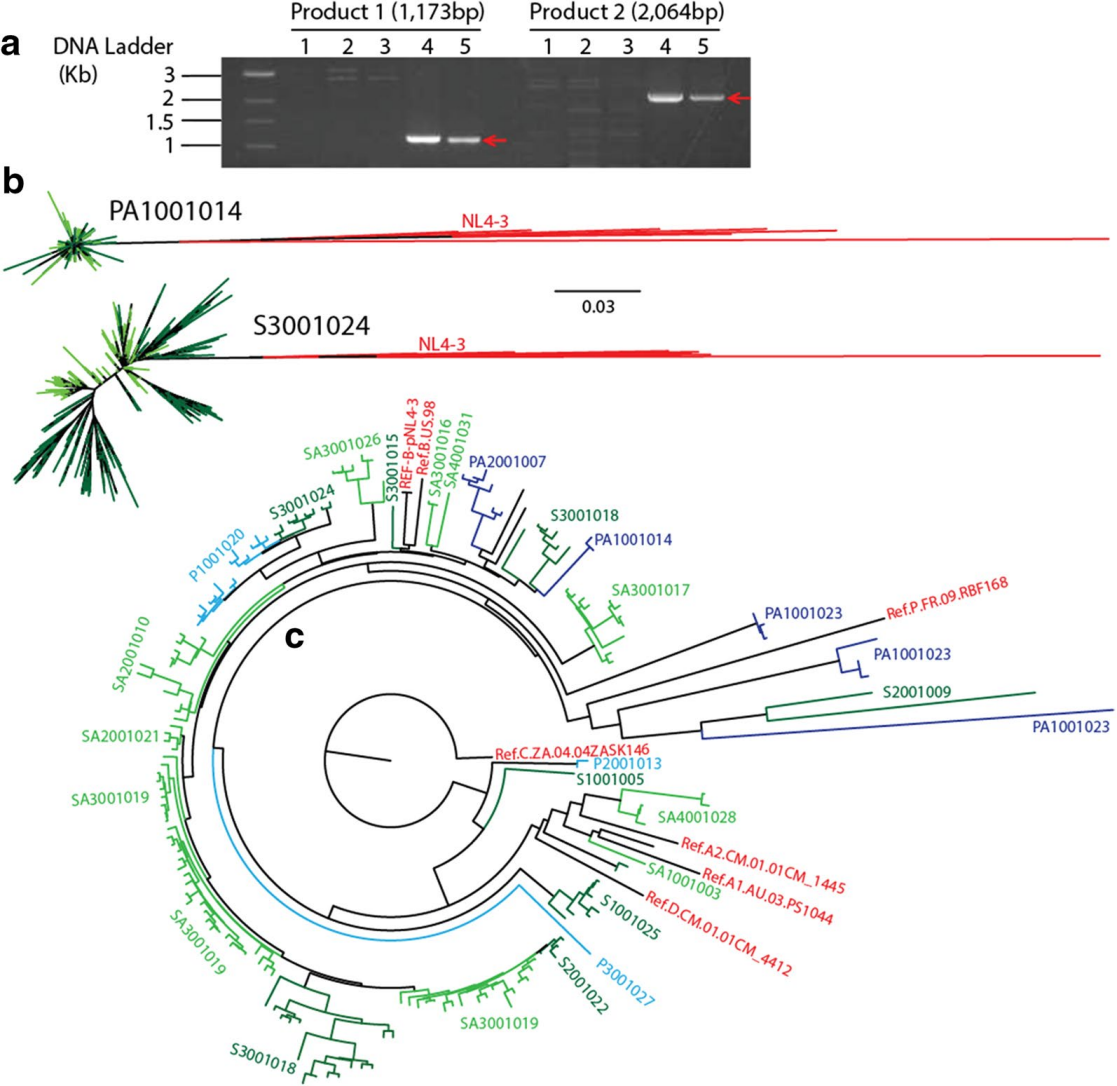
Absence of residual infectious HIV-1 in SAV001 vaccine after inactivation: Detection of HIV-1 proviral DNA in infected cells by PCR and sequence analysis of viruses isolated from HIV-1 positive volunteer study subjects by 454 pyrosequencing. *Lanes 1* Passage 3 of SAV001 vaccine virus infected cell DNA; *Lanes 2* Passage 5 of SAV001 vaccine virus infected cell DNA: *Lanes 3* Passage 10 of SAV001 vaccine virus infected cell DNA; *Lanes 4* Passage 3 of HIV-1 NL4-3 wild type virus infected cell DNA; *Lanes 5* Passage 3 *gm*HIV-1_NL4-3_ mutant virus infected cell DNA (**a**). Multisequence alignments of reads were constructed using MUSCLE (PMID: 15034147), and all phylogenetic analysis was performed with MEGA5 (PMID: 21546353). The sequence reads were used to generate phylogenetic trees for further analysis through alignment with the reference viral sequences including NL4-3, 1 or 2 strains from HIV-1 subtype A, B, C, D, and F (**b**, **c**). All numbers represent subject ID in S (SAV001 group); SA (SAV001 + Adjuvant group), P (Placebo group), and PA (Placebo + Adjuvant group). PA1001014 and S3001024 in **b** were randomly selected to show the detail data

 One hundred million CD4^+^ A3.01 human T cells were infected with either 5 × 10^7^ TCID_50_ of the wild type HIV-1_NL4-3_ virus or 5 × 10^7^ TCID_50_ of *gm*HIV-1_NL4-3_ virus, or 30 human doses (3 mg) of SAV001 vaccine with assumption that all cells will be infected at second or third passages of infected cells. Samples were incubated for 1 h for virus adsorption, culture media added, and incubated in a 37 °C CO_2_ incubator. Infected cells were split every 3 days and refurbished with fresh A3.01 cells. The infected cells were harvested at the time of each subculturing and stored at −20 °C. Infected cellular DNA was extracted and assessed by PCR using a pair of primers covering the 5300–6473 nucleotide region of HIV-1 NL4-3 genome (Fig. 3a Product 1). This region produces a 1173 bp PCR product and another pair of primers spanning 459–2523 nucleotides of HIV-1 NL4-3 genome (Fig. 3a Product 2) which will produce a 2064 bp PCR product.

### Sequence analysis of virus from HIV patients by 454 pyrosequencing

To detect any SAV001 vaccine virus specific DNA the proviral DNA in 10^6^ PBMCs from each participant was extracted with QIAamp DNA blood kit (Qiagen), with which nested PCR was used to amplify ~2.8 kb of HIV-1 *env* fragments. Pyrosequencing of the HIV-1 in plasma was performed following reverse transcription-PCR to obtain the gp120 *env* DNA amplicons. Nested PCRs of the C2–V3 regions were prepared for pyrosequencing using the external gp120 *env* products as templates. The primers E110 and E125 [26] were modified to contain the 454 adaptor sequences (Roche Lib-A Primer A and Primer B), followed by a 10 basepair Multiplex Identifier (MID) sequence at the 5′ end to permit sample pooling. These barcoded amplicons were quantified by fluorometry with the Quant-iT PicoGreen dsDNA Assay Kit (Life Technologies), pooled in equimolar concentrations, and sequenced on a 454 GS Junior System (Roche Diagnostics) using the GS Junior Titanium Sequencing chemistry. The resulting reads were trimmed to exclude the MIDs and primer sequence, and low-quality reads were filtered using the GS Run Processor according to length and quality scores.

The proviral DNA in 10^6^ PBMCs from each of the participant HIV patients was extracted with QIAamp DNA blood kit (Qiagen), with which nested PCR was used to amplify ~2.8 kb of HIV-1 *env* fragments. The external primers were envA (forward, HXB2 numbering nt5954–5982, 5′-GGCTTAGGCATCTCCTATGGCAGGAAGAA-3′)/envN (backward, nt9145-9171, 5′-CTGCCAATCAGGGAAGTAGCCTTGTGT-3′), and the internal primers were envB (forward, nt6202–6228, 5′-AGAAAGAGCAGAAGACAGTGGCAATGA-3′)/envM (backward, nt9068–9096, 5′-TAGCCCTTCCAGTCCCCCCTTTTCTTTTA-3′). The PCR amplifications were performed with 5 μl of extracted proviral DNA and the following conditions: 98 °C 2 min, [98 °C 10 s, 63 °C 30 s, 72 °C 2 min] × 35 cycles, and 72 °C 10 min. The PCR products were purified with a QIAquick gel extraction kit (Qiagen). To perform the 454 pyrosequencing, we used the following primers: E110 (forward, nt7002–7025, 5′-CTGTTAAATGGCAGTCTAGCAGAA-3′) and E125 (backward, nt7315–7338, 5′-CAATTTCTGGGTCCCCTCCTGAGG-3′) with 66 different barcodes at the 5′ ends of the both primers. Amplicons were purified using the Agencourt AMPure XP (Beckman Coulter) magnetic bead PCR purification system, quantified using the Quant-iT PicoGreen dsDNA Assay Kit (Life Technologies), and then equalized to a concentration of 10 ng/μl. Samples were pooled into two tubes to prevent redundancy of barcoded primer used for the amplification of samples. Amplicons were added to the emulsion at one copy per bead. EmPCR was done according to the Roche EmPCR manual, and Amplicon sequencing was performed according to the Roche Amplicon sequencing manual. Sequences were processed and collected using Roche GS FLX System software. Following sequencing collection, sequences were extracted according to barcode by the Java script Extraction and sorted by barcode into a folder containing two different files. The resulting reads were trimmed to exclude the MIDs and primer sequence, and low-quality reads were filtered using the GS Run Processor according to length and quality scores. The sequence reads were used to generate phylogenetic trees for further analysis through alignment with the reference viral sequences including NL4-3, 1 or 2 strains from HIV-1 subtype A (A1.AU.03.PS1044_Day0.DQ676872; A2.CM. 01.01CM_1445MV.GU201516), B (B.US.98.1058_11.AY331295), C (C.ZA.04.04ZASK146.AY772699), D (D.CM.01.01CM_4412HAL.AY371157), and F (F1.FR.96.96FR_MP411.AJ249238; F2.CM.02.02CM_0016BBY.AY371158), as well as CRF07-BC (0.07_BC.CN.05.XJDC6431_2.EF368372) and CRF08-BC (08_BC.CN.97.97CNGX_6F.AY008715).

### Recognition of cell surface HIV-1 trimeric gp120 by anti sera from vaccinated subjects

To evaluate recognition of trimeric HIV-1 envelope glycoproteins (Env) by the elicited antibodies, CEM.NKr cells infected with full-length pNL4.3 GFP ADA Env (WT) or deleted for *nef*- (N-) and *vpu* (U-) were stained at 48 h post-infection with 1/1000 dilution of HIV+ sera from SAV001 + Adjuvant, SAV001, Placebo + Adjuvant, or Placebo subjects and then labeled with an Alexa-Fluor-647 conjugated anti-human IgG secondary antibody and analyzed by flow cytometry [27, 28].

### Assessment of safety

Adverse event (AE), local reaction, clinical laboratory, vital signs, physical examinations, and plasma HIV-1 RNA levels were assessed. Reporting began with the administration of the study vaccine (Day 1) and continued until completion of study visit at Day 365. An AE was defined as any unfavorable and unintended sign, symptom, or disease temporally associated with the study vaccine, whether or not it was considered to be related to the vaccine. Subjected, observed, and elicited AEs were recorded using the terminology from the Medical Dictionary for Regulatory Activities. Investigators assessed the severity of each AE according to the DAIDS Table for Grading the Severity of Adult and Pediatric Adverse Events. If an AE occurred that was not graded by these criteria, its severity was evaluated as mild, moderate, severe, and potentially life threatening. The causality/relationship between the study vaccine and the AE was assessed as definite, probable, possible, and unlikely depending on the symptom.

A serious AE (SAE) was defined as any untoward medical occurrence, if it resulted in death or was life threatening, required inpatient hospitalization or persistent or significant disability. Pregnancy was not considered as a SAE, but all pregnancies that occurred within 90 days were reported by the investigator and subjects were discontinued from the study.

Each subject was instructed to complete a diary card for 7 days following study vaccine administration, to describe local reactions, systemic reactions, and other selected indicators of reactogenicity. If a reaction continued beyond 7 days after vaccination, it was recorded as an AE. Blood and urine samples were collected for hematology, clinical chemistry and urinalysis, respectively.

Plasma HIV-1 RNA was quantitated by the Roche COBAS^®^ Ampliprep/COBAS^®^ TaqMan^®^ HIV-1 test, V2.0 (Roche Molecular Diagnostics, California). If the subject was suspected to have an increase in plasma HIV-1 RNA, he/she was slated for an unscheduled visit within 2–4 weeks after the occurrence and retested plasma HIV-1 RNA.

### Assessment of immunogenicity

The immunogenicity of the vaccine was assessed using the following humoral response and T-cell counts. The response was evaluated by assessment of antibody titers against p24, p17, gp120, and gp41 by standard ELISA.

Total CD3^+^ T cell count/μl, as well as CD4^+^ T-cell count/μl and CD8^+^ T-cell count/μl were quantified in peripheral blood by flow cytometry, and percentages of CD3^+^ CD4^+^ T-cells, and CD3^+^ CD8^+^ T-cells were calculated.

### Assessment of neutralizing antibodies

Neutralization activity was measured using a luciferase-based assay in TZM-bl cells as described at the protocol from Montefiori Laboratory, Duke University [29, 30]. The assays were performed with HIV-1 tier 2 PRB926-04.A9.4237 (subtype B) [31], tier 1 HxB2. DG (subtype B) [32], 93UG065 (subtype D from Uganda)^NIH ARP^ and Q168ENVa2 (subtype A) [33] ENV-pseudotyped viruses. Serum samples were heat-inactivated to destroy complement by incubation at 56 °C for 1 h before use. Because anticoagulants in plasma are toxic to the cells at low plasma dilutions, and in our experiment we found more than tenfold dilution did not influence the cell growth, so samples were first diluted by 1:10 followed by threefold serial dilutions in the 96-well plate, with Britelite (Perkin Elmer) using a Victor V plate reader (Perkin Elmer). The value of more than 50% reduction in relative luminescence units (RLU) compared to the control was determined as positive.

### Statistical analysis

Due to the limited sample size of this study, it was likely that most inferential analyses would not be meaningful. Therefore, overall trends are reported. Continuous data were summarized by the group using descriptive statistics and categorical data by the group using frequency tables (frequencies and percentages).

## Results

### The *gm*HIV-1_NL4-3_ used in SAV001 vaccine retained the intact virion structure and is immunogenic

The construction of the genetically modified *gm*HIV-1_NL4-3_ virus is depicted in Fig. 1a–c and the sequences of the *gm*HIV-1_NL4-3_ genome have been confirmed by RT-PCR using honeybee melittin-specific primer with modified *nef* gene-specific primer (Fig. 1d). We examined the morphology of killed whole *gm*HIV-1_NL4-3_ virions in SAV001 by transmission electron microscopy. Virions in SAV001 remain intact after all purification and inactivation processes (Fig. 1e). The presence of HIV-1 p24 capsid protein and docking glycoprotein gp120 on the purified *gm*HIV-1_NL4-3_ virus particles was confirmed by Western blot (Fig. 1f) and by aggregation of human T-cells in culture (Fig. 1g–j). In addition, SAV001 was found to be immunogenic in naïve rats. Intramuscular administration of SAV001 induced anti-gp120 antibody in rat as verified by ELISA titer (Fig. 1k, l). Results from these experiments provide clear evidence that purified and inactivated *gm*HIV-1_NL4-3_ virus used in SAV001 vaccine retained the intact virion structure including surface glycoprotein gp120.

### The *gm*HIV-1_NL4-3_ virus used in the SAV001 vaccine is completely inactivated

The complete inactivation of HIV-1 to generate a killed whole-HIV-1 vaccine is the most critical step to develop a killed whole-virus vaccine. We utilized two inactivation methods to effectively kill the *gm*HIV-1_NL4-3_ virus. The treatment of infected culture fluid with 2.5 mM of AT-2 inactivates approximately 5 logs of *gm*HIV-1_NL4-3_ virus. The AT-2 treated viruses were then exposed to 30 KGy (3,000,000 rads) of γ-irradiation (Table 1). Based on concentration of virus particles and the inability to infect C8166 cells, we estimated a minimal 10.5 log reduction in viral infectivity per 0.25 ml of SAV001 vaccine. We conducted further experiments to determine whether or not SAV001 vaccine had any residual infectious *gm*HIV-1_NL4-3_ virus particles. We infected 10^7^ A3.01 human T-cells with 30 human doses (3 mg) of SAV001, passaged these killed virus-exposed (infected) cells 10 consecutive times, isolated infected cell DNA, and tried to detect any HIV-1 proviral DNA genomic sequences by PCR using HIV-1_NL4-3_ virus specific primers. We did not find any HIV-1 NL4-3 proviral DNA sequences in SAV001 vaccine infected A3.01 cells (Fig. 3a). To further confirm the genetic mutation and safety of SAV001 vaccine, we attempted to reverse transcribe-PCR amplify the SAV001-specific viral RNA in the plasma of subjects using a primer specific for the honeybee melittin signal peptide coding sequence (Fig. 1d). As a control, we amplified a 960 bp product from viral RNA extracted from the *gm*HIV-_1NL4-3_ virus preparation but could not detect any of this viral RNA in the plasma of subjects. We then RT-PCR amplified the C2–V3 Env region of all participants before and for 4 weeks after vaccinations and analyzed the HIV-1 diversity/strain identity by 454 pyrosequencing [26]. A total of 71,338 HIV-1 C2–C3 (326 bp) *env* sequence reads were obtained from the 58 plasma samples of 29 subjects (Fig. 3b). Most importantly, all sequence reads were participant-specific and we did not identify a *gm*HIV-1_NL4-3_ viral sequence in the >70,000 sequence reads by Next Generation Sequencing. These findings further confirm the lack of detectable *gm*HIV-1_NL4-3_ virus replication within SAV001 vaccinated subjects (Fig. 3b). We concluded from these experiments that the *gm*HIV-1_NL4-3_ virus used in SAV001 vaccine is completely inactivated.

### The SAV001 vaccine is safe and well tolerated

There were no reports of potentially life-threatening AEs, SAEs, deaths, or AEs leading to study termination after vaccination in all groups. The most frequently reported System Organ Classes (SOCs) were GI disorders, infections and infestations, and musculoskeletal and connective tissue disorders. The treatment groups were comparable with regards to intensity of AEs, and the majority of events reported were mild. Overall, subjects from SAV001 + Adjuvant and Placebo + Adjuvant reported more AEs than subjects from SAV001 and Placebo. The adjuvant, Montanide^®^ISA51VG, is well-characterized, water-in-oil emulsion adjuvant. There were no differences observed between groups with regards to relationship of AE to study drug (Table 3). The majority of events were assessed as not/unlikely related to SAV001.

**Table 3:** Adverse reactions caused by SAV001 vaccination

	Group 1				Group 2			
	SAV001 (n = 13)		Placebo (n = 4)		SAV001 + A (n = 12)		Placebo + A (n = 4)	
	n (%)	Number of AEs^a^	n (%)	Number of AEs^a^	n (%)	Number of AEs^a^	n (%)	Number of AEs^a^
Any AE	9 (69.2)	17	2 (50.0)	2	9 (75.0)	30	4 (100.0)	13
Any serious AE	0	0	0	0	0	0	0	0
AE by maximum intensity^b^								
Mild	6 (46.2)	12	2 (50.0)	2	6 (50.0)	23	3 (75.0)	12
Moderate	2 (15.4)	4	0	0	3 (25.0)	7	1 (25.0)	1
Severe	1 (7.7)	1	0	0	0	0	0	0
Potentially life threatening	0	0	0	0	0	0	0	0
AE by relationship^c^								
Not related	6 (46.2)	13	1 (25.0)	1	6 (50.0)	24	2 (50.0)	11
Unlikely	2 (15.4)	3	1 (25.0)	1	2 (16.7)	5	1 (25.0)	1
Possible	1 (7.7)	1	0	0	0	0	1 (25.0)	1
Probable	0	0	0	0	0	0	0	0
Definite	0	0	0	0	1 (8.3)	0	0	0

For each level of subject summarization, a subject was counted once if the patient reported 1 or more events. Adverse events were coded using MedDRA, Version 15.1. Percentages were calculated using the number of patients in the Safety Set within each treatment as the denominator. Adverse event (AE), local reaction, clinical laboratory, vital signs, physical examinations, and plasma HIV-1 RNA levels were assessed from the administration of the study vaccine (Day 1) and continued until completion of the study visit at Day 365. An AE was defined as any unfavorable and unintended sign, symptom, or disease temporally associated with the study vaccine, whether or not it was considered to be related to the vaccine. A serious AE (SAE) was defined as any untoward medical occurrence, if it resulted in death or was life threatening, required inpatient hospitalization or persistent or significant disability

*AE* adverse event

^a^Number of AEs = the total number of AEs at each level of summarization

^b^Subject was counted once according to the maximum intensity experienced if the patient reported 1 or more AEs

^c^Subject was counted once according to the most related if the patient reported 1 or more AEs. An AE (diarrhea) for Patient 3001018 in SAV001 group was originally considered to be possibly related to study drug. Follow-up communication, however, indicated that the event occurred when the patient took his antiretroviral medication on an empty stomach. Given the updated information, the investigator revised his assessment of the event to be unrelated to study drug. Because the follow-up communication occurred subsequent to the database being locked, the value in the database was not revised

The majority of subjects reported no local or systemic reaction immediately after vaccination.

The most frequently reported local or systemic reactions in daily diary cards for 7 days after administration were mild or moderate muscle pain, joint pain, tiredness, and other feelings of discomfort. In SAV001 and Placebo, muscle pain reactions were not identified on Day 7 although 15.4% of subjects reported moderate muscle pain on Day 1. Within SAV001 + Adjuvant and Placebo + Adjuvant, the incidence of muscle pain was higher in subjects of SAV001 + Adjuvant than Placebo + adjuvant on Days 1 and 2; however, this pattern reversed on Days 3 through 7. Only one subject in the Placebo + Adjuvant experienced an increase in muscle pain severity from mild on day 1 to severe joint pain on Day 7. No subjects reported swelling, rash, oral temperature ≥38 °C, redness or skin hardening at the injection site.

Based on the above data, it is safe to conclude that SAV001 + Adjuvant did not cause any serious local, systemic reactions, or adverse events. Therefore the study vaccine is tolerable for use in humans.

### The SAV001 vaccine elicited humoral immune responses against the HIV vaccine components p24/p17/gp120 and gp41

**Fig. 4:**
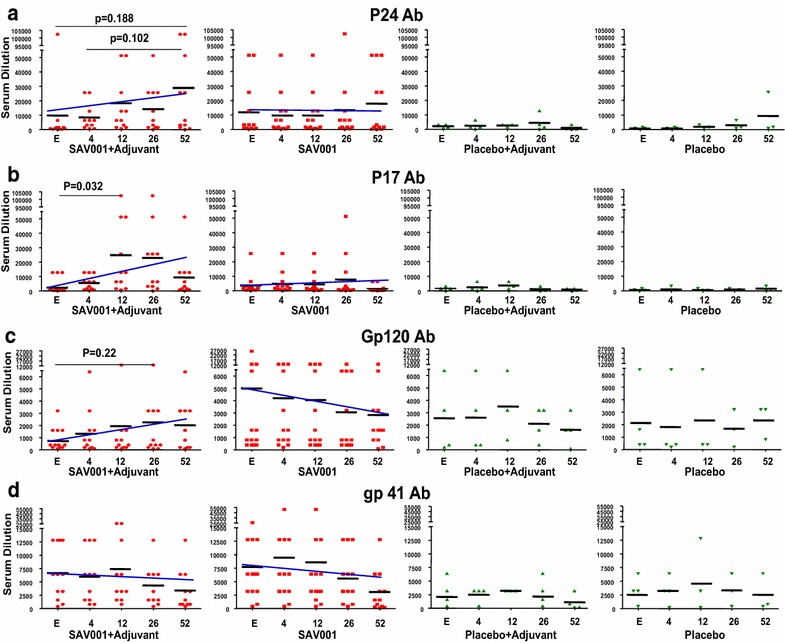
Humoral immune responses of SAV001 vaccine. The humoral immune response was evaluated by assessment of antibody titres against p24, p17, gp120, and gp41 by ELISA. Microtiter plates were coated with specific HIV-1 recombinant antigens (p24, p17, gp120, and gp41 derived from HIV-1 IIIB (Immuno Diagnostics, USA) and serial dilutions of subject sera reacted against the specific antigens. The plates were read on the plate reader at 450 nm. Levels of antibodies in subjects from the entry and from week 4 to week 52 after vaccination were analyzed; p24 antibody (**a**), p17 antibody (**b**), gp120 antibody (**c**), and gp41 antibody (**d**). Numbers in the x-axis represent entry (*E*) and weeks after vaccination

Despite that the main goal of the current study was to evaluate the safety of our inactivated vaccine in humans, we also evaluated some elicited humoral immune responses. High antibody titers against p24, p17, gp120, and gp41 were observed in all the subjects at baseline as expected, considering all the enrolled subjects were infected with HIV-1 and on cART. The SAV001 + Adjuvant group had increased antibody titers against p24, p17, and gp120 proteins from baseline at all time points. In contrast, the SAV001 alone (without adjuvant) group showed insignificant increase of antibodies against HIV-1 proteins (Fig. 4a–d).

**Fig. 5:**
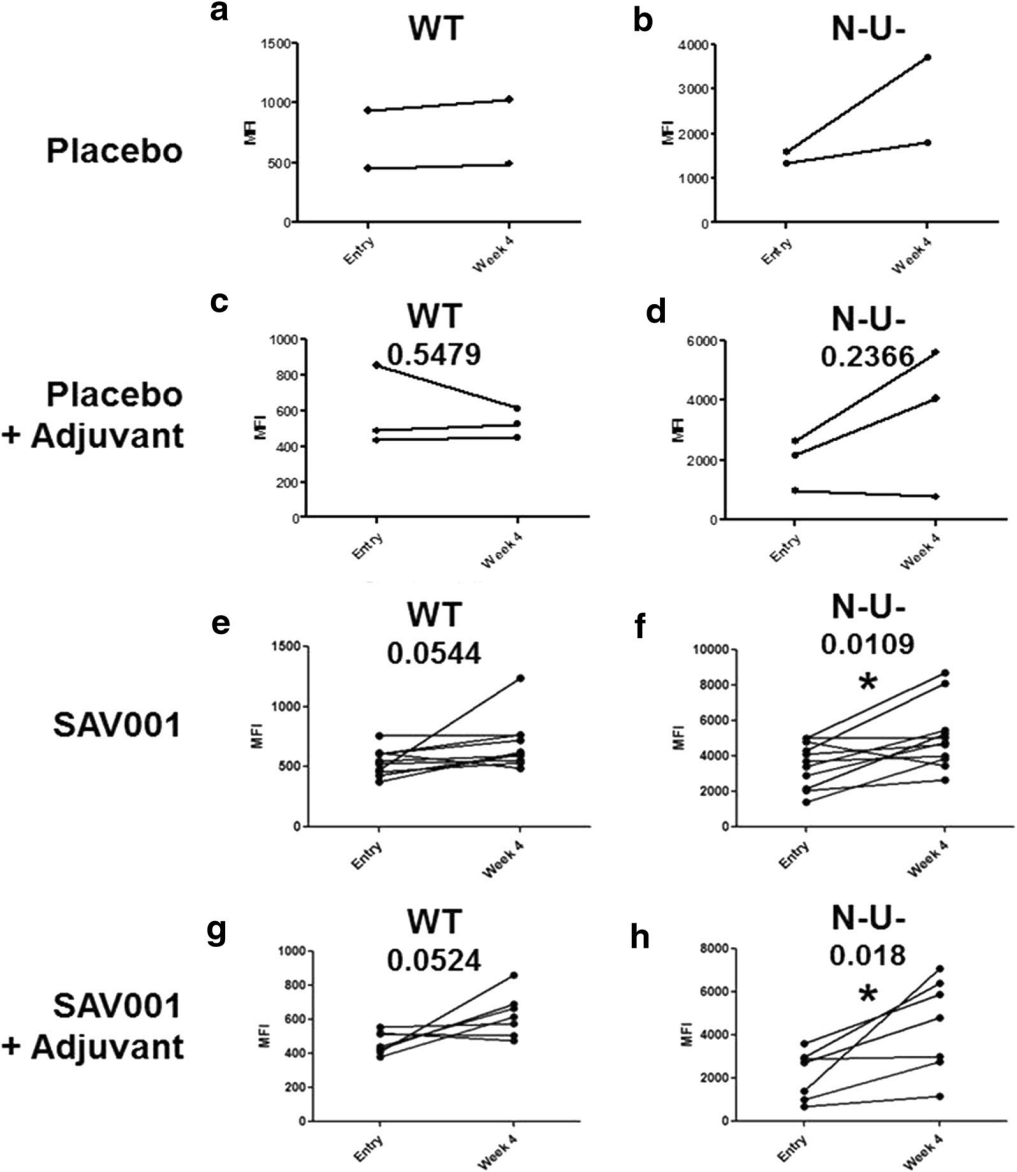
SAV001 elicits antibodies that recognize HIV-1 envelope glycoproteins at the surface of infected cells. CEM.NKr cells infected with full-length NL4.3 GFP ADA Env (WT) (**a**, **c**, **e** and **g**) or deleted for *nef* and *vpu* (N-U-) (**b**, **d**, **f** and **h**) were stained at 48 h post-infection with 1/1000 dilution of HIV + sera from Placebo- (**a** and **b**), Placebo + Adjuvant- (**c** and **d**), SAV001- (**e** and **f**) or SAV001 + Adjuvant- (**g** and **h**) vaccinees and then fluorescently labeled with an Alexa-Fluor-647 conjugated anti-human IgG secondary Ab [27, 28, 36]. Shown is the mean fluorescent intensity (MFI) of staining by sera from study participants. Statistical significance was tested using a paired *t* test (*p < 0.05)

Antibodies binding to monomeric HIV-1 gp120 are a poor surrogate for virus neutralization or clearance within vaccinated individuals [34]. Thus, we also measured binding of antibodies in plasma to CD4^+^ T cells expressing trimeric HIV-1 Env glycoproteins on the cell surface using flow cytometry [35]. Plasma from subjects immunized with SAV001 with/without adjuvant had antibodies with enhanced recognition (p = 0.0544 in SAV001, p = 0.0524 in SAV001 + Adjuvant) of the native trimeric Env protein as compared to the plasma from placebo subjects (p = 0.5479 in SAV001 + Adjuvant) (Fig. 5). Interestingly, this trend became statistically significant (p = 0.0109 in SAV001, p = 0.018 in SAV001 + Adjuvant) when we measured recognition of Env protein at the surface of Nef- Vpu- HIV-1 infected cells where trimeric Env glycoprotein engages with surface CD4 and exposes CD4-induced epitopes [27, 28, 36].

### Augmented neutralizing antibodies in response to SAV001 in a number of vaccine recipients

**Fig. 6:**
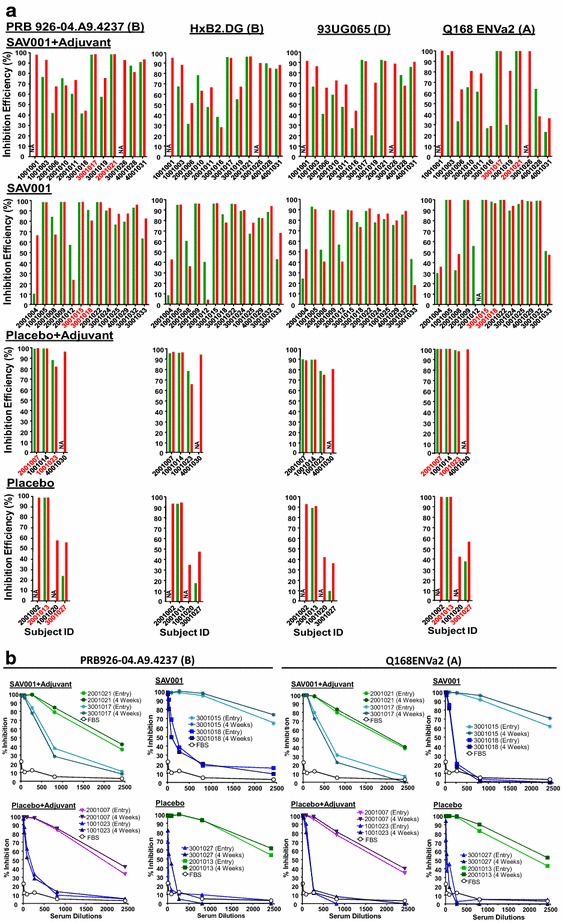
Neutralizing antibody response after vaccination. The analysis of the neutralizing antibodies before (*green bar*) and 4 weeks after (*red bar*) vaccination were carried out with 1:30 dilutions of sera and luciferase reporter gene expression was determined with Britelite (Perkin Elmer) using a Victor V plate reader (Perkin Elmer) (**a**). Neutralization activity of serial dilutions of high neutralizing antibodies of participants’ sera (*red* numbers on Fig. 5a) at both pre- and post-vaccination, using PRB926-04.A9.4237 (subtype B) and Q168ENVa2 (subtype A). Each data point represents the average IC50 of a single serum sample tested with the either virus, and the data from pre- and post-vaccination samples were paired for comparison (**b**). *NA* not available

It is believed that a truly efficacious HIV vaccine should be able to harness the highly desired broadly neutralizing antibody response. Although the primary objective of this Phase I human clinical trial was an evaluation of the safety of a killed whole-HIV-1 vaccine, we tested the levels of neutralizing antibodies in plasmas of the vaccinated and control participants who are HIV positive and on cART. We used the standard TZM-bl (NIH AIDS Reagent Program) assay [29, 30] to determine neutralizing antibody titers when infecting with HIV-1 tier 2 PRB926-04.A9.4237 (subtype B) [31], tier 1 HxB2.DG (subtype B) [32], 93UG065 (subtype D)^NIH ARP^, and Q168ENVa2 (subtype A) [33] Env-pseudotyped viruses. A rapid analysis of the neutralizing antibodies pre- and 4 weeks post-vaccination were carried out with 1:30 dilutions of participants’ sera and luciferase reporter gene expression was determined with Britelite (Perkin Elmer) using a Victor V plate reader (Perkin Elmer). We found approximately 50% of subjects’ sera in SAV001 + adjuvant and SAV001 without adjuvant showed an elevated neutralizing antibody level against HIV-1 subtypes B, D, and A (Fig. 6a). Some subjects’ sera contain high levels of neutralizing activities. In order to determine the level of neutralizing antibodies in samples showing close to 100% neutralization, dilutions of participants’ plasma (from 1/10 to 1/2430) were made and neutralization activity was measured through the reduction in luciferase reporter gene expression in TZM-bl cells following a 48-h incubation period with a single round of virus infection (Fig. 6b). As expected, the enrolled volunteers, even before vaccination with SAV001, showed high levels of neutralizing antibody titres. Sixteen out of 27 completely tested subjects had neutralizing antibodies against the subtypes B, D, and A pseudotyped viruses at entry (Fig. 6). After a single-dose SAV001 vaccination, 5 out of 10 study subjects in SAV001 + Adjuvant group showed elevated neutralizing antibody titres against HIV-1 B, D, and A subtypes (Fig. 6). Five out of 13 subjects’ plasma from the SAV001 without adjuvant showed elevated neutralizing antibodies against subtype B, and 6 out of 13 subjects showed elevated neutralizing antibodies against subtypes D and A. Similarly, Placebo + adjuvant group and Placebo group also showed some neutralizing antibodies at 4 weeks after vaccination. The level of elevated antibody titres were not related to the viral loads (only three subjects showed 20–50 copies of viral RNA/ml) nor the changing in CD4 cell counts (data not shown).

## Discussion

This randomized, observer–blinded, placebo–controlled Phase I study was designed to assess safety and tolerability as primary end-points, and humoral immunogenicity as a secondary end-point of a killed whole-HIV-1 vaccine, SAV001. To our knowledge, this is the first human clinical trial with a killed whole-HIV-1 vaccine and the results of this study were highly encouraging. The single intramuscular administration of 100 µg of the SAV001 with or without adjuvant was safe and well tolerated in the studied cohort, as there were no potentially life-threatening AEs, SAEs, deaths, or AEs leading to study termination in any of the 33 enrolled subjects.

The greatest concern in the killed whole-virus vaccine strategy for HIV is whether the virus is completely inactivated. This concern for safety would impede any large scale production of the vaccine which is necessary to decrease the cost and the widespread use of HIV vaccine application, especially in developing countries. In the present study, we succeeded in generating a safe inactivated vaccine suitable for mass manufacture. To further ensure the safety of the vaccine, we deleted the *nef* gene for attenuation, and used both chemical (AT-2) and physical (γ-irradiation) inactivation to provide complete killing. The test for in vitro replication of SAV001 using the most sensitive methods showed that virus replication was absent even after 10 consecutive passages of human T lymphocytes after exposure (infection) to a large amount of the vaccine. Furthermore, following immunization, highly sensitive, external-nested PCR amplifications using vaccine-specific primers on the viral RNA in plasma confirmed the lack of any vaccine strain virus genome. In contrast, the external-nested PCR amplification by our generic HIV-1 primer sets was able to amplify HIV-1 RNA despite undetectable viral loads by the less sensitive Roche Amplicor assay (<50 copies/ml). Pyrosequencing of these PCR products before and after immunization with SAV001 again confirmed the lack of HIV-1 NL4-3 in over 70,000 HIV sequence reads and the presence of only patient-specific HIV-1 strains. These results provide very strong evidence that the technologies applied in this study completely inactivated the HIV-1 and ensured the safety of the vaccine recipients.

Despite the complete inactivation of *gm*HIV-1_NL4-3_ virus used in this present study, we only enrolled HIV-1 positive asymptomatic volunteers based on discussions with the US FDA. Therefore, even though the study is designed for safety and tolerability evaluation, the nature of the study limited our ability to assess the immunology of vaccine formulations. Previous studies have shown that HIV-1 infection elicits antibody responses to proteins encoded by HIV-1 *gag, pol* and *env* genes, and the antibody response to various proteins appears at different stages of infections [37]. As expected, most of the subjects in our study had a high baseline antibody titer against viral structural proteins. However, with a single SAV001 intramuscular vaccination, the humoral immune response was significantly increased by boosting secondary anti-HIV antibody responses in vaccinated groups suggesting a strong immunogenicity of the SAV001 vaccine.

A major goal of HIV-1 vaccine research is the design of immunogens capable of inducing protective levels of broadly neutralizing antibodies that bind to the viral envelope glycoprotein and neutralize the infectivity of HIV-1 [21]. Earlier reports described monoclonal antibodies, including a pair of somatic variants that neutralized over 90% of circulating HIV-1 isolates [38, 39]. The importance of the design of immunogens, especially the trimeric form of the envelope glycoproteins, capable of inducing broadly neutralizing antibodies is the major focus of the current HIV-1 vaccine research [40, 41]. Even though our killed whole-*gm*HIV-1_NL4-3_ virus was purified and completely inactivated through combined chemical and physical inactivation processes, its Env glycoproteins were not readily shed and were still functional (Fig. 1e–l). Our results suggest that SAV001 vaccine will mimic natural infection through its native viral structure, especially the native form of envelope glycoprotein which is crucial for eliciting broadly neutralizing antibodies. Indeed, when compared to placebo control, our data supported this hypothesis as SAV001 was able to stimulate anti-gp120 antibodies in plasma that could recognize trimeric Env glycoproteins at the surface of infected cell. Although most of subjects were infected with HIV-1 subtype B in our trial (Table 1), sera from these subjects were able to neutralize not only subtype B but also subtypes A and D which is consistent with the notion that HIV-1 superinfection is a limited event [42–45] particularly those who have been infected with HIV-1 for more than several months. Thus, HIV-1 vaccine based on one subtype may be able to protect against infections of other subtypes. The immune response studies in HIV-negative subjects in our Phase II clinical trial may reveal this possibility. Furthermore, we tried to correlate the level of viral loads with the level of neutralizing antibodies in order to understand why many participants showed elevated neutralizing antibody titres at 4 weeks after vaccination in all four groups. Only three subjects showed detectable levels of viral loads (20–50 copies/ml), with no correlations between the viral loads and the level of neutralizing antibodies.

## Conclusion

SAV001, the genetically modified and killed whole-HIV-1 vaccine, is safe and well tolerated after a single intramuscular injection. The combination of chemical and physical inactivation procedures is adequate to completely kill the genetically modified HIV-1 NL4-3 virus while retaining the function and immunogenicity of viral proteins. Vaccination with SAV001 could enhance humoral immune responses including broadly neutralizing antibody production in HIV-1 negative individuals. Therefore, SAV001 represents a promising starting point for development of a safe and effective HIV-1 vaccine using the killed whole virus approach.
